# Packed Red Blood Cells Are an Abundant and Proximate Potential Source of Nitric Oxide Synthase Inhibition

**DOI:** 10.1371/journal.pone.0119991

**Published:** 2015-03-20

**Authors:** Charles F. Zwemer, Robertson D. Davenport, Juan Gomez-Espina, Elisa Blanco-Gonzalez, Steven E. Whitesall, Louis G. D'Alecy

**Affiliations:** 1 Department of Biology, Dickinson College, Carlisle, PA, United States of America; 2 Pathology Department, University of Michigan Medical School, Ann Arbor, MI, United States of America; 3 Department of Physical and Analytical Chemistry, University of Oviedo, Oviedo, Spain; 4 Department of Molecular and Integrative Physiology, University of Michigan Medical School, Ann Arbor, MI, United States of America; Emory University, UNITED STATES

## Abstract

**Objective:**

We determined, for packed red blood cells (PRBC) and fresh frozen plasma, the maximum content, and ability to release the endogenous nitric oxide synthase (NOS) inhibitors asymmetric dimethylarginine (ADMA) and monomethylarginine (LNMMA).

**Background:**

ADMA and LNMMA are near equipotent NOS inhibitors forming blood’s total NOS inhibitory content. The balance between removal from, and addition to plasma determines their free concentrations. Removal from plasma is by well-characterized specific hydrolases while formation is restricted to posttranslational protein methylation. When released into plasma they can readily enter endothelial cells and inhibit NOS. Fresh rat and human whole blood contain substantial protein incorporated ADMA however; the maximum content of ADMA and LNMMA in PRBC and fresh frozen plasma has not been determined.

**Methods:**

We measured total (free and protein incorporated) ADMA and LNMMA content in PRBCs and fresh frozen plasma, as well as their incubation induced release, using HPLC with fluorescence detection. We tested the hypothesis that PRBC and fresh frozen plasma contain substantial inhibitory methylarginines that can be released chemically by complete *in vitro* acid hydrolysis or physiologically at 37°C by enzymatic blood proteolysis.

**Results:**

*In vitro* strong-acid-hydrolysis revealed a large PRBC reservoir of ADMA (54.5 ± 9.7 µM) and LNMMA (58.9 ± 28.9 μM) that persisted over 42-d at 6° or -80°C. *In vitro* 5h incubation at 37°C nearly doubled free ADMA and LNMMNA concentration from PRBCs while no change was detected in fresh frozen plasma.

**Conclusion:**

The compelling physiological ramifications are that regardless of storage age, 1) PRBCs can rapidly release pathologically relevant quantities of ADMA and LNMMA when incubated and 2) PRBCs have a protein-incorporated inhibitory methylarginines reservoir 100 times that of normal free inhibitory methylarginines in blood and thus could represent a clinically relevant and proximate risk for iatrogenic NOS inhibition upon transfusion.

## Introduction

**Fig 1:**
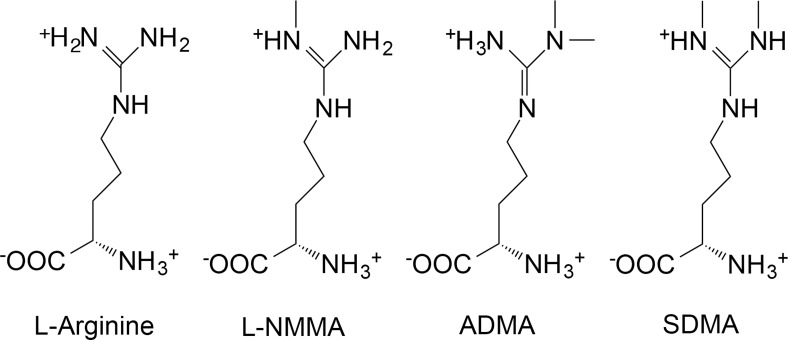
Arginine and its endogenous methylated derivatives. Arginine is the normal substrate for NOS resulting in NO formation. A single methylation of arginine produces monomethylarginine (LNMMA) which, along with asymmetric dimethylarginine (ADMA), are endogenous inhibitors of NOS. ADMA and LNMMA are hydrolyzed by dimethylarginine dimethylaminohydrolase (DDAH). Symmetric dimethylarginine does not inhibit NOS. These structures were drawn as they exist at mammalian physiological pH using ChemDraw software (PerkinElmer Informatics) and data from PubChem at NCBI at the National Library of Medicine (USA).

Endogenous inhibition of nitric oxide synthase (NOS) is linked to clinically relevant, dose-dependent pathologies such as ischemic vasoconstriction [1], platelet aggregation [2], and myeloperoxidase release [3]. The ability of asymmetric dimethylarginine (ADMA) and monomethylarginine (LNMMA) (Fig. 1) to inhibit all isoforms of NOS is firmly established [4], as is the function of NOS to produce NO. Historical nitro-vasodilators such as nitroglycerin and sodium nitroprusside were linked by Murad’s group [5] to endothelial derived relaxing factor and subsequently to NO mediated endothelial vascular relaxation [6] establishing central physiological and pathophysiological roles for NOS and for this study of potential NOS-inhibition. This pathway involves free arginine as the normal substrate for NOS producing NO and both ADMA and LNMMA as near equipotent competitive (K_i_ ~ 1 μM) endogenous inhibitors of NOS [4]. Specifically, Leiper and Vallance described the IC_50_ values for L-NMMA and ADMA for NOS (all three forms) as being “approximately equipotent” [7] and “on the order of 2 to 5 μM”. Tsikas et al., stated, “ADMA and NMA (L-NMMA) inhibit NO synthesis with comparable potencies *in vitro* and *in vivo*, in blood vessels, and macrophages in animals and in man.” [8], while Cardounel et al., used a combination of cell culture work, vascular reactivity studies and *in vivo* carotid artery injury techniques [4] to reveal the K_i_ of ADMA and L-NMMA to be 0.9 and 1.1 μM respectively. Both ADMA and LNMMA are primarily cleared from the blood by hydrolysis by dimethylarginine dimethylaminohydrolase (DDAH) [9, 10] and to a lesser degree by the kidneys.

Blood is a bulk carrier of ADMA and LNMMA and we propose that blood may also act as their primary physiological source. The presence, concentrations, and release potential of these inhibitors in commercially available packed red blood cells (PRBC) and fresh frozen plasma, in contrast to fresh blood and plasma, is unknown. Both inhibitors are found free in plasma (< 0.5 μM) and are widely incorporated in proteins in fresh animal (43 μM)[11] and human whole blood (36 μM) [12]. Total molar concentration of these amino acid inhibitors can be determined by strong acid hydrolysis to individual amino acids. The potentially releasable store of NOS inhibitors can be calculated by subtraction of the free-in-plasma concentration of ADMA and LNMMA from the total concentration (including incorporated). It follows that when the total store of ADMA and LNMMA is many (100X) times that of plasma, then the ongoing normal proteolysis of even a small fraction of the store will add to the systemic circulating burden of NOS inhibition. The specific tissues or proteins that serve as the origin for elevated circulating ADMA and LNMMA have yet to be defined for PRBC's and fresh frozen plasma.

The point of this study was to investigate the impact of storage on PRBC inhibitory methylarginine total content. PRBCs are commercially available, derived blood products, separated by centrifugation and size exclusion techniques and then mixed with a large number of preservation agents. They are kept under non-physiological conditions in polyvinylchloride bags made flexible with phthalate esters [13], have a finite shelf life, and thus they are different from fresh whole blood. We determined if stored PRBCs and fresh frozen plasma could serve as a direct source of ADMA and LNMMA by virtue of containing proteins that incorporate pathologically significant quantities of these releasable inhibitory methylarginines. Once subjected to proteolytic breakdown, these proteins would release ADMA and LNMMA into solution (in plasma) and be available for inhibition of NOS. In high blood product use areas in critical care, many seemingly disparate injury modalities (trauma, multiple organ failure, and sepsis) utilize shared, leukocyte-based inflammatory-pathologic pathways [14, 15]. This elevated and often ongoing inflammatory state could provide the conditions necessary for enhanced proteolytic release of these NOS inhibitors. Specifically, we wanted to determine the theoretical maximum capacity of these blood products to release free inhibitory methylarginines and thus their ability to contribute the total NOS inhibitor burden. However, in this study we did not address the eventual *in vivo* (following transfusion) formation and release of inhibitory methylarginines nor their likely deleterious effects on NO bioavailability. We did, however, clearly establish a potential and previously unidentified risk of NOS inhibition following PRBC transfusion.

## Materials and Methods

Methods briefly described below are largely based upon our previous publications [3, 11, 12, 16]. Detailed descriptions are found in S1 Methods.

### Blood components

All blood components (PRBC and fresh frozen plasma) were obtained from the University of Michigan Health System Blood Bank, originated from the American Red Cross Blood Services, Southeastern Michigan Region, and were stored in accordance with AABB Standards or experimentally frozen at -80°C to completely inhibit normal enzymatic activity. Each PRBC unit was Adenine—Saline Added, Leukocytes Reduced, Group O, and Rh positive, from different male donors.

### Ethics Statement

A University of Michigan Human Research Protection Program Institutional Review Board approval was not required for use of these de-identified commercial blood products.

### Strong Acid hydrolysis of blood proteins

Upon arrival and at 2, 4, and 6 weeks, well-mixed PRBC or fresh frozen plasma were sampled using aseptic technique and acid hydrolyzed to release previously formed inhibitory methylated arginines [17] yielding total ADMA and LNMMA (i.e. the sum of free and protein-incorporated). This is thus a direct measurement of the total ADMA and LNMMA in the PRBC or fresh frozen plasma samples. Samples were kept on ice and diluted with an ice-cold solution containing NG-monoethyl-L-arginine monoacetate salt (MEA) as HPLC internal standard [18]. 100 μL aliquots of 40-fold diluted PRBC or 2-fold diluted fresh frozen plasma were dried in a centrifugal evaporator (GeneVac, EZ-Bio), placed in a hydrolysis chamber (Picotag, Waters, Milford, MA) containing 6 N HCl (constant boiling), and incubated *in vacuo* at 150°C for 1h and re-dried. These protein hydrolysates (free amino acids) were re-suspended in 100 μL of 20 mM HCl and supernatant was separated and analyzed by HPLC with fluorescence detection. Values were corrected for dilution and normalized to assayed hemoglobin when appropriate.

### Incubation Protocols

Blood contains enzymes capable of breaking down proteins to amino acids thus liberating any incorporated methylated arginine. A 37°C water bath was used for incubation as this temperature is a reasonable approximation of *in vivo* conditions for generation of ADMA and LNMMA previously demonstrated to occur by proteolytic release in both rat [11] and human blood [12]. At multiple time points (0 to 6 wk.) after acquisition, PRBCs were lysed by three serial freeze-thaw cycles to eliminate cell membrane diffusional barriers. Lysates were centrifuged and supernatant was incubated at 37°C. Samples were taken at 0, 1, 3 and 5 h and analyzed for ADMA and LNMMA by HPLC. Fresh frozen plasma was similarly processed.

### Sample preparation for methylarginines assay and High-performance liquid chromatography

Samples (100 μL) were prepared for HPLC analysis of methylarginines and were quantified by reverse-phase liquid chromatography (Breeze System, Waters) as previously described [3, 11, 12, 16] and detailed in S1 Methods.

### Protein and Hemoglobin assay

Protein was quantified using the Pierce BCA [bicinchoninic acid] Protein Assay [Rockford, IL]. Bovine Gamma Globulin [BGG, Bio-Rad Product No. 500–0208 Hercules, CA] was used as a protein standard according to manufacturer’s instructions. Absorbance [Bio-Rad SmartSpec 3000 Spectrophotometer] of eight standards was used to create a polynomial regression to which sample absorbencies were applied to calculate protein concentrations.

Hemoglobin was quantified spectrophotometrically in alkaline hematin detergent complex (D-575 AHD) using an effective millimolar extinction coefficient of 27.08 at 575 nm per heme (ε^575^ = 6.77 ± 0.018). This method is reported by Fenchik, McFaul, and Tsonev [19] to give excellent agreement with assays based upon more traditional potassium ferricyanide and potassium cyanide (Drabkin’s Reagent) [20] methodologies and is detailed in S1 Methods.

### Catalase Evaluation as a source of ADMA in PRBC

Using the online protein database of the National Center for Biotechnology Information (U.S. National Library of Medicine), the amino acid sequence of human catalase was analyzed to determine the percent arginine content (http://www.ncbi.nlm.nih.gov/protein/NP_001743.1). The analysis revealed 29 arginine residues present in the 527 amino acid protein sequence. Thus, arginine accounts 5.5% of the sequence composition of human RBC-catalase; a well-characterized soluble red blood cells cytosolic enzyme. To determine catalase concentration in PRBC units we sampled duplicate aliquots from fresh, well-mixed PRBC bags (n = 2) and froze them for shipment. At the analytical laboratory they were thawed, aliquoted, hemolysed, and prepared for analysis using size exclusion chromatography and inductively coupled plasma mass spectrometry (ICP-MS). Technique details are in S1 Methods.

### ADMA Concentration in human catalase

Pure human catalase (Sigma Aldrich-Product Number C3556) was subjected to strong acid hydrolysis followed by HPLC direct quantification for ADMA as described above. Using data derived from catalase concentration measured in PRBCs and the ADMA concentration determined from pure catalase, we calculated the percent of total ADMA in PRBCs attributable to catalase as detailed in S1 Methods.

### Statistical analysis

Data are reported as mean ± one standard deviation (SD) and were analyzed using repeated measures two-way analysis of variance. If significant differences were detected, data were further evaluated using Tukey’s *post-hoc* multiple comparison test (α = 0.05). Incubation data were evaluated using linear regression analysis to determine the relationship between incubation time and accumulation of ADMA. Significance for all tests was accepted at p ≤ 0.05.

## Results

### Acid hydrolysis releases total-MA from PRBC’s

**Fig 2:**
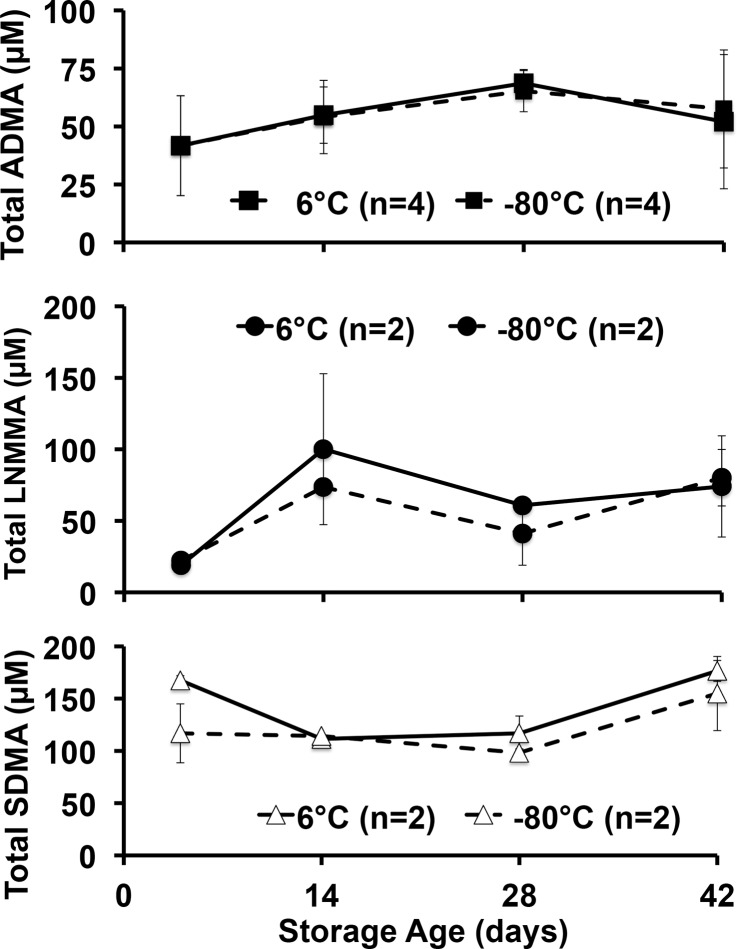
Total methylarginines obtained by strong acid hydrolysis of pre-aliquoted PRBCs stored at 6°C or -80°C. No statistically significant differences were detected between 6°C or -80°C storage at any time (pooled to 5, 14, 28 and 42 d) for ADMA, LNMMA and symmetric dimethylarginine and no change from control was detected. It is likely that neither PRMT activity nor DDAH-induced hydrolysis was a dominating effect under these storage conditions. However, equal formation and hydrolysis cannot be ruled out.

**Fig 3:**
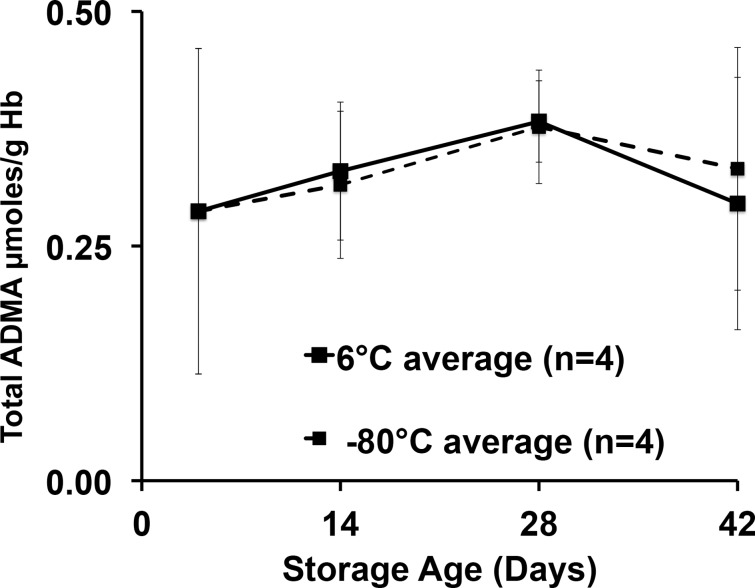
ADMA scaled against hemoglobin concentration over storage time at 6°C or -80°C. In an attempt to account for any dehydration of the samples under either storage condition (6°C or -80°C), we measured hemoglobin concentrations paired with each ADMA measurement to calculate ADMA to hemoglobin ratio. The time course and pattern remained unchanged over time suggesting no measurable desiccation effect on sample concentration over the 42-day storage.

We first determined the total NOS inhibitor content and relative stability in PRBCs over 42 days, and analyzed total (free plus protein incorporated) inhibitory methylarginines from samples stored under standard conditions (6°C) or frozen at -80°C to inhibit enzymatic activity. Upon receipt, aliquots from each unit were prepared, with four maintained at 6°C and four at -80°C to avoid repeated freeze thaw or warming cycles encountered by serial sampling at 0, 2, 4, and 6 weeks of the same sample over 42 days. Despite upward trends, no statistically significant effect of storage time or temperature was detected. Our critical observation is that we found, for the first time in PRBCs, an overall 42-day average (combined 6°C and -80°C values) for total ADMA of 54.5 ± 9.7 μM (Fig. 2). This is in contrast to 36μM found in fresh human blood reported previously [12] and is almost 100 times the free ADMA measured in these samples (0.58 ± 0.12 μM). By contrast, LNMMA averaged 58.9 ± 28.9 μM or over 700 times the free LNMMA (0.08 ± 0.02 μM). Combined, total ADMA and LNMMA, represent a maximal inhibitory methylarginines release capacity of 114 μM for hydrolyzed PRBC. The symmetric dimethylarginine average was 132 ± 30 μM over this same period. When total ADMA was normalized to g/hemoglobin (Fig. 3), the overall 42-day combined average (6°C and -80°C samples) appeared to be stable at an average (N = 8) of 0.33 ± 0.04 μmoles ADMA/g Hb. Thus lysed PRBC contain a liberated inhibitory methylarginines reservoir two orders of magnitude greater than the free inhibitory methylarginines in ready to transfuse PRBC. (Figs. 2 and 3).

### Percent ADMA attributable to catalase

On average only 1.8% of the total ADMA found in PRBCs could be attributed by calculation to the measured presence of catalase. Table 1 shows the individual hematologic values for PRBC units sampled for catalase. The average value for μmol ADMA/ g catalase was determined separately on pure human catalase following strong acid hydrolysis. This value multiplied by the measured catalase concentration in g/L PRBC gave the estimated μmol ADMA/L of PRBC.

**Table 1:** Hematologic values for PRBCs in Catalase experiment.

Sample	Hct (%)	Hb (g/L)	PRBC protein (g/L)	PRBC Catalase (g/L)	PRBC ADMA (μmol/L)	*ADMA/Catalase (μmol/g)	Catalase ADMA/PRBC (μmol/L)	% ADMA due to catalase
Unit 18.1	65.8	195.2	102.5	0.233	30.9	4.42	1.03	3.3
Unit 18.2		203.2	108.1	0.094	35.1	4.42	0.42	1.2
Unit 19.1	57.8	172.4	91.0	0.123	33.7	4.42	0.54	1.6
Unit 19.2		171.4	84.1	0.097	41.0	4.42	0.43	1.0
**Mean ±** SD	61.4 ±5.7	185.6 ± 16.1	96.4 ± 10.9	0.13 ± 0.066	35.2 ± 4.3	4.42	0.61 ±0.29	1.8 ±1.0

*The average value for μmol ADMA/ g catalase was determined separately on pure human catalase following strong acid hydrolysis. This value was multiplied by the measured catalase concentration in g/L PRBC to give the estimated μmol ADMA/L of PRBC.

### Incubation induced free-inhibitory methylarginines release from PRBC’s

**Fig 4:**
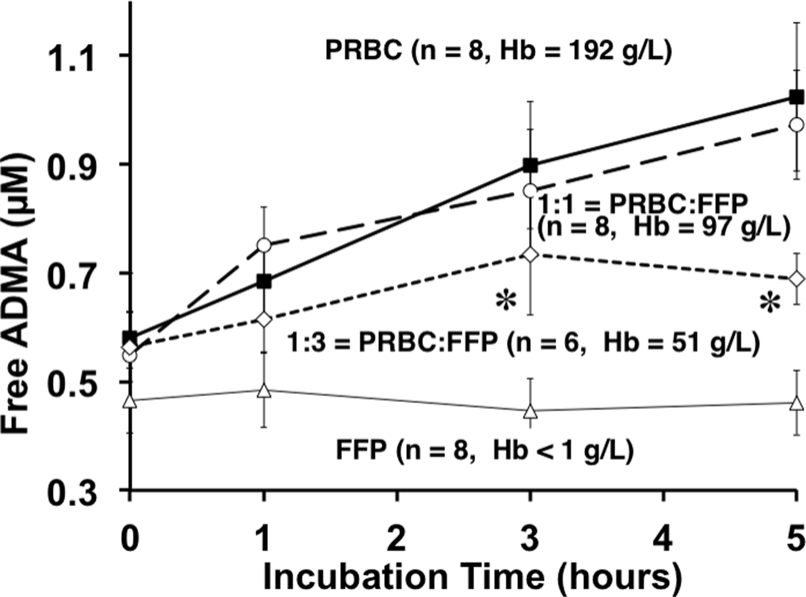
Incubation-induced release of Free ADMA over 5h by Blood Product type. Incubation of defrosted fresh frozen plasma (open triangles), supernatants of PRBC alone (solid squares), a mixture of 1:1 PRBC and fresh frozen plasma (open circles), and a mixture of 1:3 PRBC and fresh frozen plasma (open diamonds). Repeated measures Two-way ANOVA shows significant differences of ADMA among PRBC and PRBC: fresh frozen plasma groups over the incubation period (p<0.006). Tukey’s *post-hoc* multiple comparison test revealed significant differences of ADMA (α = 0.05) among groups at individual sampling points. At three and five hour time points, ADMA in PRBC and 1:1 PRBC: fresh frozen plasma was significantly (*) higher than 1:3 PRBC: fresh frozen plasma. Hemoglobin measurements confirmed PRBC concentration and dilutions.

We observed that 5h incubation of PRBC supernatant nearly doubled free ADMA in solution (Fig. 4). Linear regression analysis revealed a positive, time-dependent accumulation of ADMA in PRBCs and 1:1 mixture of PRBC: fresh frozen plasma (p = 0.0072 and p = 0.0388 respectively). These PRBC-data are similar to, but distinct from, our initial observations of a similar release response based upon fresh freeze thawed rat whole blood [11] and fresh freeze thawed human blood [12]. This PRBC-based observation is also different from that of Teerlink’s group [21] who used fresh washed erythrocytes. However, the important consistency to note is that incubation at 37°C effectively released inhibitory methylarginines from PRBCs, whole blood, washed red blood cells or red blood cell lysates in each of these studies.

When fresh frozen plasma was thawed and incubated under identical conditions that resulted in marked release of ADMA from PRBCs, we found (Fig. 4) no statistically significant increase (open triangles) in released free ADMA. To simulate transfusion practice we also incubated equal parts PRBC supernatant mixed with thawed plasma (open circles) expecting a 50% reduction in ADMA release due to fresh frozen plasma dilution of the PRBC. To our surprise, the 1:1 mixture yielded near identical concentrations of free ADMA. Assuming that, within this 1:1 incubation mixture, the fresh frozen plasma fraction released no ADMA, then the 50% diluted PRBC supernatant portion must have released the same quantity of ADMA as the 100% PRBC solution. That is, twice the free ADMA was released from half the PRBCs. For this to happen either more substrate was available to react in the 1:1 mixture and or greater hydrolytic activity was at work during this 5h period. We have not determined which mechanisms are responsible for this relative increased release but do note the potential added NOS inhibitor burden possible with such a response. We confirmed the 1:1 dilution by measuring the hemoglobin concentration, which was halved (97 g/L vs. 192 g/L original PRBC), and thus the other components of the PRBC supernatant would also be halved. The 1:3 (PRBC to fresh frozen plasma) dilution markedly attenuated the ADMA release response (open diamonds) and suggests a trailing off of the release at five hours perhaps due to depletion of substrate for proteolysis.

**Fig 5:**
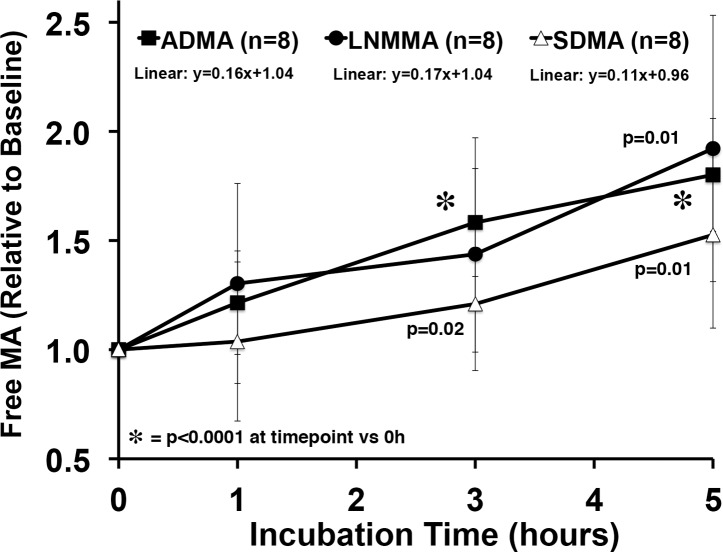
Relative methylated arginine release over 5 h from PRBC incubation. All three methylated arginines, ADMA, LNMMA, and symmetric dimethylarginine were released in proportionately similar amounts during the 5h incubation at 37°C. The starting free concentrations were different (0.58 ± 0.12, 0.08 ± 0.02, and 0.20 ± 0.12 respectively). Free ADMA, LNMMA and symmetric dimethylarginine increased significantly from baseline by 5h. Linear regression analysis failed to detect differences among methylated arginines over 5h.

The baseline (Time = 0) steady-state PRBC concentrations of free ADMA (0.58 ± 0.12 μM), symmetric dimethylarginine (0.20 ± 0.12 μM), and LNMMA (0.08 ± 0.02 μM) were different so, we normalized each to their time zero baseline value and calculated response to incubation as a fraction of baseline (Fig. 5). The ADMA data are the same as in Fig. 4 except expressed as a fraction of baseline. The slope of the normalized release line is near identical for ADMA and LNMMA while the symmetric dimethylarginine slope tends to be lower but is not statistically different. Given that LNMMA and ADMA have been reported to be near equipotent NOS inhibitors [4] this robust release represents a significant source for circulating ADMA and LNMMA.

## Discussion

### Potential Risk of NOS Inhibition Following PRBC Transfusion

In this study we report two novel and important findings: 1) fresh PRBCs contain a very large reservoir of incorporated (bound) endogenous NOS inhibitors (Figs. 2 and 3); and 2) 37°C incubation releases significant free amounts of both ADMA and LNMMA (Figs. 4 and 5) into the supernatants regardless of the PRBC storage age at the start of incubation. Both observations indicate that PRBC’s used in transfusion have the capacity to increase the NOS inhibitory burden in the recipient following transfusion even if only <1% of bound (40 to 70 μM) ADMA and LNMMA were released as further discussed below.

The existence of blood borne endogenous NOS inhibitors is not new, however, demonstrating that PRBC transfusion products contain and have the capacity to release pathologically relevant quantities of both ADMA and LNMMA is novel. This suggests a previously unanticipated risk of PRBC transfusions. While normal control of vascular NOS activity by circulating endogenous NOS inhibitors may have an important physiological role, excessive inhibition of ubiquitous endothelial Nitric Oxide Synthase (eNOS), has the potential to increase post-transfusion morbidity and mortality due to dysregulation of the critical vasodilator NO.

We examined PRBC and fresh frozen plasma as possible protein sources of ADMA and LNMMA due to blood’s proximity to eNOS containing vascular endothelial cells. It is illustrative to consider circulating blood as a large (~5 Kg) organ (or continually renewed reservoir) in intimate contact with endothelial cells containing eNOS. When proteolysis of blood proteins releases significant quantities of ADMA and LNMMA these endogenous inhibitors would have almost immediate access to eNOS for inhibition.

### ADMA and LNMMA Combined eNOS Inhibitor potential

Both ADMA and LNMMA were consistently present in fresh PRBC and throughout the storage period and when added together present a 114 μM (55 + 59 μM) concentration of endogenous NOS inhibitor potential. This is the first report of large quantities of LNMMA in addition to ADMA in PRBC’s and thus identifies the maximum NOS inhibitory potential ever reported. Physiological or pathophysiological release mechanisms, even amplified in the critically ill, are not likely to ever approach this maximum release concentration. However, Teerlink’s group [22] by prolonging the incubation period to 18h increased the total ADMA released to 60% of total incorporated. If, following transfusion, as little as 0.5 to 1.0% of the incorporated amino acids were released by ongoing proteolytic breakdown it would double the plasma concentration in each unit administered. In a multi-unit transfusion scenario, pathological plasma levels would rapidly be attained in the transfusion recipient. Adding a similar concentration of the total LNMMA (~59μM) from the PRBC's would double the potential combined NOS inhibitory potential of a PRBC transfusion.

### Total content of inhibitory methylarginines in stored PRBC at 6° and -80°C vs. fresh human blood

We first reported fresh whole blood from rats (43 μM) [11] contained significant incorporated and hence total ADMA and subsequently showed that fresh whole blood from normal human volunteers contained 36 μM [12]. Similar findings in humans have been recently confirmed by Teerlink's group, however they reported 15 μM ADMA incorporated in washed red blood cells that were frozen at -20°C overnight and re-suspended in two volumes of water prior to analysis [22]. Distinct from these reports based on whole blood, the total ADMA in PRBC’s in the current study (Fig. 2) ranged from 42 μM on day four to 69 μM on day 28 of standard storage conditions. This could reflect the greater red blood cell concentration (density) in these PRBC samples compared to fresh whole blood. Had we similarly diluted our PRBC sample our higher measured ADMA concentrations would have been diluted to approximately 18 μM, which is in reasonably close agreement with Teerlink's group. However, in our study it is unclear what effects the blood draw and blood bank processing involved in preparing PRBCs might have had on increasing the inhibitory methylarginines in the PRBC samples. For this study we did not have access to pre-donation whole blood samples to determine if they contained even lower inhibitory methylarginines averages that would have been even closer to what the Teerlink’s group obtained using fresh washed and diluted human blood. It is important to note that neither our laboratory nor Teerlink’s report repeated paired measurements of the total ADMA and LNMMA in freshly drawn erythrocytes and then again in these same samples once they are prepared for clinical use as PRBC. Such a paired analysis of fresh donor blood (prior to donation) and once processed to PRBC’s would be required to determine if processing itself increased the total inhibitory methylarginines content in the PRBCs.

It is also possible that protein arginine methyltransferase (PRMT) was active during storage at 6°C, but data collected from the stored samples at 6° compared to -80°C (Figs. 2 and 3), suggest that there was either little or no ongoing PRMT activity contributing to the methylated arginine pool, or that the methylated residues were eliminated at the same rate as they were formed (e.g., by DDAH activity). Allowing that the 6° and -80°C total ADMA values were indistinguishable (Figs. 2 and 3) it is most likely that neither methylation nor DDAH hydrolysis occurred during storage at either temperature. This does not, however, exclude the possibility of protein modifications during storage that would predispose PRBC proteins to subsequent hydrolysis once the product was introduced by transfusion into a critically ill patient’s elevated proteolytic milieu.

### Erythrocyte catalase as a potential source of ADMA

The Tsikas group using GC-MS/MS has shown that neither hemoglobin [23] nor albumin [24] contain significant amounts of ADMA. Thus, we looked for red blood cell proteins that had a high concentration of arginine residues. We measured red blood cell catalase protein in an attempt to identify a dominant intra-erythrocyte ADMA reservoir. The extensive human red blood cells proteome is not entirely characterized, so we chose catalase due to its relatively high arginine content and its high intracellular solubility. Additionally, Yokoro et al.[25] detected ADMA in carbonic anhydrase II and catalase in Sprague-Dawley rat RBC. Our data (please see Table 1) reveal that erythrocyte catalase only accounts for a small fraction (1.8%) of total ADMA available in PRBC. The NCBI analyses of arginine-rich erythrocyte proteins suggest that superoxide dismutase and cytoskeletal proteins such as ankyrin and spectrin may also contribute as methylarginines reservoirs, but these remain untested. Please see the “S1 Methods” section titled "Methods for Evaluation of PRBC catalase as a source of ADMA" for further discussion.

### Free vs. Incorporated ADMA and LNMMA

The plasma concentration of free ADMA alone is traditionally used to reflect the capacity of vascular NOS inhibition, yet it is also widely accepted that LNMMA (present at lower concentrations) is a nearly equipotent NOS inhibitor [4]. We submit that combining the free circulating ADMA plus LNMMA concentration, for a total inhibitory methylarginine concentration, more accurately represents the systemic NOS inhibitory burden. It is possible that LNMMA's role has been under appreciated since the historical, widespread use of LNMMA as an internal HPLC standard has obviated reporting on its presence in plasma or protein hydrolysates as an analyte of interest. We have eliminated this shortcoming from this study by using NG-monoethyl-L-arginine monoacetate salt (MEA) as our internal HPLC standard. This permits assessment of the total and free LNMMA in addition to the ADMA in blood products. While the normal plasma levels of LNMMA are very low (0.08 μM) the potential releasable pool is very high (58.9 μM) and similar to that of ADMA.

Mechanistically, it is the pool of free ADMA and LNMMA circulating in plasma that is readily available to move by Y+ facilitated diffusion into vascular endothelial cells and inhibit NOS [26]. Y+, or cationic amino acid transporters are present in both vascular endothelial [27] and red blood cells [28] and support bidirectional movement of L-arginine and methylarginines between intra and extracellular compartments [29]. The overall plasma steady state inhibitor concentration then is a function of the balance between addition to and removal from this volume. The presumptive pathway for addition is posttranslational methylation of protein-incorporated arginine by PRMT [7]. Once arginine is methylated, the protein itself must then be hydrolyzed to release the ADMA and LNMMA into the pool, as only free amino acids can compete with arginine to inhibit NOS. Removal of inhibitory methylarginines from the pool is more clearly defined and is primarily (~83%) driven by the action of DDAH [9, 10], while only a minor fraction of the pool is cleared by the kidneys [30, 31].

### Removal of inhibitory methylarginines from plasma pool

DDAH activity plays a major role in *in vivo* [9, 10] ADMA hydrolysis. Indeed, numerous laboratories have attempted to address modulating the balance between formation and removal of ADMA and LNMMA by identification of DDAH analogs as potential therapeutic derivatives to reduce plasma ADMA concentrations by increased or restored ADMA hydrolysis. Thus, restoring or enhancing removal [32, 33] is a strategy for managing various pathological states with elevated plasma ADMA and LNMMA.

It is less clear however whether DDAH hydrolysis is active *in vitro*, during storage or incubation [21]. In our PRBC incubation protocols, no attempt was made to modify any ongoing DDAH activity. However, our previous work with rat blood [11] demonstrated a robust, presumably DDAH-like activity during incubation when the samples were spiked to elevate starting ADMA levels. In addition, 4124W (DDAH inhibitor) [34] or supplemental Zn [35] decreased the disappearance of added ADMA further suggesting the presence of DDAH-like activity. It is likely that in those studies the elimination of ADMA was more evident because the higher substrate (ADMA) concentration was driving the ADMA hydrolysis reaction. In the present study it is possible that there was some undetected, ongoing DDAH-like activity that was obscured by simultaneous release of “new” ADMA into solution. However, here we present no evidence that human PRBCs contain DDAH—agreeing with a similar recent observation by the Teerlink group [21, 22] using fresh human blood. While Kang [36, 37] reported the presence of DDAH in human blood that hydrolyzed both ADMA and LNMMA, Teerlink’s group reported no increase in citrulline in incubated erythrocytes and they interpreted this as indicative of no DDAH activity [22]. It is possible, however, that any citrulline resulting from the DDAH hydrolysis of ADMA and LNMMA was simply converted back to arginine. Our current data do not resolve this issue and ADMA-spiked PRBC supernatants would need to be evaluated to provide adequate resolution.

### Balance of proteolytic release and elimination

The balance of normal rates of proteolytic release and subsequent elimination by hydrolysis produce normal plasma concentrations for ADMA and LNMMA in humans, [38] rats, and mice [39] that are sub-micromolar (0.1 to 1.0 μM). In multiple ischemic cardiovascular diseases including stroke[40], coronary artery disease [41], and peripheral arterial occlusive disease[42], the pathological plasma levels of ADMA and LNMMA are higher and well within range of their endothelial NOS inhibitory constants (K_i_, 0.9 μM and 1.1 μM respectively [4]) thus establishing conditions necessary for NO dysregulation.

### Release of inhibitory methylarginines *in vitro* by strong acid hydrolysis vs. 37°C incubation

In our initial observation of freeze-thawed (lysed) rat whole blood (FTWB) we reported that strong acid hydrolysis liberated a total of 43 μM ADMA and incubation for 5h at 37°C released enough free ADMA to increase its free concentration from 0.95 μM to 2.32 μM or more than 2.4 times baseline—yet we detected no ADMA change when fresh rat plasma alone was incubated [11]. In the same study, protease inhibitor cocktails markedly attenuated ADMA release suggesting a proteolytic release mechanism. In patients with end stage renal disease and matched hypertensive control subjects with normal renal function, we demonstrated similar plasma levels to those found in rats for both total ADMA and incubation-induced release of ADMA [12].

Teerlink’s group has confirmed both the presence of protein incorporated ADMA in fresh washed human erythrocytes and, using a similar incubation protocol, induced release of ADMA that was attenuated by protease inhibitors [21]. In separate studies, serum albumin was found to contain 0.3 nmol ADMA/g albumin while hemoglobin was reported to contain none [23, 24]. More recently Teerlink’s group [22] extended the incubation-release protocol to 18h and demonstrated a sustained ADMA release that could account for 22% at 2h and 60% at 18h of the total content released in their washed fresh erythrocyte preparation. While these studies point to the erythrocyte as a significant source of ADMA and LNMMA, what was missing was an assessment of the total content and release capability of commercial blood products (PRBC and fresh frozen plasma). Thus, they too could not account for changes that might have occurred during sample preparation and storage of red blood cells and plasma for transfusion. The magnitude of the release-response they report is comparable to what we observed. This suggests that the mechanisms for release withstand a variety of experimental manipulations and processing procedures. By extension, these likely also remain intact in the PRBC product used for clinical transfusion.

### Inhibitory methylarginines in fresh frozen plasma

It appears that regardless of the species or method of preparation, erythrocytes are capable of releasing significant quantities of endogenous NOS inhibitors. By contrast, incubation of thawed fresh frozen plasma alone at 37°C for 5h, failed to show any increase in free ADMA (Fig. 3) supporting our initial observation using fresh rat plasma [11]. In this study, we initially concluded that plasma either does not have the methylated proteins to be hydrolyzed or does not contain active proteases to release the ADMA and LNMMA. A 1:1 dilution of PRBC with plasma reduced the “PRBC-protein” but did not reduce overall release rates for ADMA. Either additional substrate must have been available to react in the 1:1 mixture and/or greater hydrolytic activity was at work during this 5h period. Because incubation of plasma alone did not release inhibitory methylarginines it is most likely that plasma did not contain active proteolytic enzymes at the time of incubation. We therefore conclude that the 1:1 mixture simply supplied additional inhibitory methylarginines containing proteins for the PRBC-based proteolytic enzymes to hydrolyze. Perhaps if we diluted the PRBC supernatant with normal saline the release of ADMA would have been proportionately reduced. Thus we conclude both PRBC and plasma contain MA-substrates but only the PRBC appear to have active proteolytic enzymes necessary for NOS inhibitor release from blood proteins.

### Potential Adverse Consequences of Excess NOS Inhibition

In the vasculature, NO is formed by eNOS [43, 44] and is a naturally occurring, highly reactive, and widely distributed signaling molecule. Endothelial cells contain NOS and their failure to produce NO is widely associated with vascular endothelial dysfunction. Direct damage to endothelial cells and or competitive inhibition of eNOS by ADMA or LNMMA reduces the tissue and plasma concentration of NO and this contributes to cardiovascular pathologies characterized as endothelial dysfunction [45, 46]. Understanding the determinants of plasma ADMA and LNMMA concentrations is essential to understanding endothelial dysfunction that, in turn, is integral to understanding much of the pathophysiology of cardiovascular disease.

Mihout et al used an 8-week ADMA infusion in normal mice and induced glomerular and renal vascular fibrosis with elevated collagen I & II and fibronectin consistent with the development of atherosclerosis, cardiovascular disease and progression of renal disease [1]. Elevated ADMA has been attributed to its impaired systemic clearance by DDAH, and overexpression of DDAH reduces ADMA [47–49] attenuating pathological changes in otherwise normal mice. We previously demonstrated short-term (60 min) ADMA infusion (0.0125 mg*Kg^-1^*min^-1^) in humans significantly increased plasma myeloperoxidase (MPO) a NO-oxidizing hemoprotein with proinflammatory properties [3]. Richir et.al. linked onset of organ failure to a significant increase in both mean arterial pressure and systemic vascular resistance and decrease in cardiac output with acute ADMA infusions (increased plasma ADMA from 0.52 to 3.3 μM). Arginine depletion via arginase coupled with ADMA infusion in the same study magnified these increases [50]. Together, such studies point to a direct, causal role for elevated NOS inhibition by ADMA in cardiovascular dysfunction. It is also possible that the loss of NO availability described by both groups is due instead to increased release of endogenous inhibitors of NOS (ADMA and LNMMA) given the elevated levels of proteolysis associated with hemolysis. In addition hemoglobin scavenging of NO and RBC-derived arginase depletion of arginine (substrate of NOS) have been identified as additional contributors to endothelial dysfunction.

### Potential Hemolysis-dependent Disruption of NO Homeostasis by Hemoglobin

Gladwin and Kim-Shapiro made a compelling argument that storage lesions might be explained by NO dysregulation attributable to impaired NOS activity. They focused on the interaction of free hemoglobin with NO causing hemolysis-dependent disruption of NO homeostasis, increased NO catabolism and loss of NO-generating function [51, 52]. Stamler’s group published data [53–55] on the acute loss of S-nitrosohemoglobin (SNO-Hb) within 3h of collection. Such a loss would be anticipated to result in a loss of NO bioavailability; perhaps more importantly, they suggested that SNO-HB repletion might improve transfusion efficacy. We agree that loss of NO availability and freed hemoglobin impairs NOS homeostasis and thus contributes to storage lesions. Data from this current study show that the endogenous NOS inhibitors, ADMA and LNMMA, are present in high concentrations during storage and thus could produce a protracted impairment of NOS activity when infused as a bolus and could produce ongoing NOS inhibition by continued release *in vivo* from fragile red blood cell disruption and MPO release [3]. We anticipate that this accumulation of endogenous NOS inhibitors during storage and continuing once infused would contribute more strongly to a sustained NOS dysfunction and thus the overall adverse outcomes of transfusion of aged blood products. We anticipate that infused NOS inhibition by ADMA and LNMMA is thus at a minimum complementary to increased NO catabolism in causing the storage lesion.

### Potential Arginase Contribution to NOS Inhibition

Arginase eliminates arginine and is thus a possible additional contributor to reduced NO formation. The limited availability of arginine would thus reduce NOS activity during storage. Bernard and co-workers [56] demonstrated increased arginase activity in a leukoreduced red blood cell preparation. The addition of nor-N-ω-OH-L-arginine, an irreversible arginase blocker, decreased arginase activity. Reduced NOS substrate could reduce NO production. However, when these units are infused they mix with the recipient’s normally abundant circulating arginine. Thus, while it is likely that reduced free arginine could be an issue for NO formation during storage, it is less apparent that the problem would persist upon transfusion. Instead, we propose an exaggerated endogenous NOS inhibition due to a significant accumulation of both ADMA and LNMMA during storage. In a transfusion, ADMA and LNMMA would enter the recipient's blood and endothelial cells to inhibit eNOS. The transfused ADMA and LNMMA would reduce NO production, and ongoing ADMA and LNMMA release would sustain this inhibition. However, testing endothelial dysfunction in patient infusion protocols was beyond the scope of this study.

### Limitations of this study

To reduce baseline variability, we restricted our protein source to either fresh frozen plasma from male donors or Red Blood Cells, Adenine—Saline Added, Leukocytes Reduced, Group O, and Rh positive, from male donors. Even with these restrictions, the baseline oxidant milieu of the donor blood was unknown and hence not factored into the analysis. Using only male donors allowed us to avoid potential sex-dependent differences in protease profile or proteolytic background in donor blood but none of the other likely modulators of protein turnover were assessed. In addition, when we incubated fresh human male blood, we found it had significantly greater ADMA release than that of females [12]; so, sex-dependent differences need further evaluation in controlled clinical trials assessing the endogenous NOS inhibitory status of the blood donor.

This study has not attempted to demonstrate the adverse consequences of NOS inhibitor infusion nor quantitate the NOS inhibitor burden once the blood product is infused. Instead, it demonstrates the substantial NOS inhibitory concentration of products used in transfusion medicine. We have demonstrated that PRBC have the potential to release two orders of magnitude greater than normal quantities of the endogenous NOS inhibitors ADMA and LNMMA. These inhibitory methylarginines are incorporated in blood proteins and at normal body temperatures are released from PRBCs by proteolysis. When PRBCs are infused, we would anticipate at least a transient increase in endothelial dysfunction and reduced plasma NO availability, if not frank cardiovascular pathology. In a healthy individual, aggressive inhibitory methylarginine clearance by endogenous DDAH might mitigate this hazard. However, in individuals with high proteolytic stress, or elevated proinflammatory cytokines, the incremental burden of infused ADMA and LNMMA via a PRBC transfusion could pose a significant and previously unidentified cardiovascular risk.

## Supporting Information

S1 Methods File contains detailed methodology of methylarginine sample preparation and HPLC separation and detection, hemoblobin measurement, and PRBC catalase evaluation. (DOCX)
