# Supplementary material for: Packed Red Blood Cells Are an Abundant and Proximate Potential Source of Nitric Oxide Synthase Inhibition
Source: PLoS One. 2015 Mar 20;10(3):e0119991. doi: 10.1371/journal.pone.0119991 (PMC4368738; doi:10.1371/journal.pone.0119991)
Supplement: S1 Methods — (DOCX) [file pone.0119991.s001.docx]

**Supporting Methods:**

**Methods for HPLC sample preparation for methylarginines assay**

Samples (100 µL) were prepared for HPLC analysis of methylarginines using the method previously described [[11](#_ENREF_11),[12](#_ENREF_12),[16](#_ENREF_16)]. Briefly, NG-monoethyl-L-arginine monoacetate salt (MEA) was added as the internal standard and proteins were removed by acid precipitation (1% 5-sulfosalicylic acid) on ice followed by centrifugation. The acid hydrolyzed samples do not have residual proteins but the same procedure was followed to optimize the chromatographic comparisons between samples. MEA replaces the earlier use of LNMMA as an internal standard because the previous use of LNMMA as an internal standard by us [[11](#_ENREF_11),[12](#_ENREF_12)] and others [[57](#_ENREF_57)] obviated the quantification of LNMMA as a contributing NOS inhibitor. Thus the use of MEA adds significant power to our HPLC assay since it allows for *independent* quantification of both known endogenous NOS inhibitors, ADMA and LNMMA. Supernatants were loaded onto preconditioned columns for solid phase extraction and eluents were dried and derivatized (AccQ*Fluor, Waters, Milford, MA).

**Methods for High-performance liquid chromatography**

Inhibitory methylarginines and symmetric dimethylarginine were quantified by reverse-phase liquid chromatography (Breeze System, Waters) as previously described [[3](#_ENREF_3),[11](#_ENREF_11),[12](#_ENREF_12),[16](#_ENREF_16)]. Separation was done using an analytical column (Waters, XterraMS C18) and guard column controlled at 36°C. Standards and samples were injected (Waters, 717 Plus Autosampler) and fluorescent peak height and area were evaluated (ex 250 nm and em of 395 nm) (Waters, 2475 Multi-wavelength Fluorescence Detector). Concentrations within each sample set were determined from a standard curve and peak height ratio using MEA as an internal standard. Average sub-µM inhibitory methylarginines detection limits (MDL) were calculated from three sets of 10 standard replicates. Coefficients of variation (inter-assay 1.7% and intra-assay 2.8%) were determined with plasma quality controls (4 replicates) included within each sample set [[12](#_ENREF_12)].)

**Methods for Hemoglobin assay**

Hemoglobin in all samples was quantified spectrophotometrically in alkaline hematin detergent complex (D-575 AHD) using an effective millimolar extinction coefficient of 27.08 at 575 nm per heme (ε^575^ = 6.77 ± 0.018). This method is reported by Fenchik, McFaul, and Tsonev [[19](#_ENREF_19)] to give excellent agreement with assays based upon more traditional potassium ferricyanide and potassium cyanide (Drabkin’s Reagent) [[20](#_ENREF_20)] methodologies. It has been further described and evaluated by Zander et al, evaluated [[58](#_ENREF_58)], and compared [[59](#_ENREF_59)] well to the Drabkin’s method [[60](#_ENREF_60)]. The AHD reagent contains 2.5 % Triton X-100 (virtually peroxide free) in 0.1 mol/L NaOH. 992 µL or 920 µL of AHD was combined with either 8 µL or 80 µL of sample (or water as blank set to 100% transmittance) in semi-micro style methacrylate cuvettes (Fisherbrand, 14-955-128, Hanover Park, Illinois, USA), covered and gently mixed by inversion five times. Each sample was processed in triplicate and values obtained were averaged. Percent transmittance was recorded (Bausch & Lomb, Spectronic 20 or Bio-Rad SmartSpec 3000 Spectrophotometer) and converted to absorbance for calculations and factoring of dilutions for final hemoglobin concentrations in g/dL.)

**Methods for Evaluation of PRBC catalase as a source of ADMA**

Catalase is a well-characterized, soluble red blood cell cytosolic enzyme that mitigates potential cytotoxic injury from partially reduced oxygen species. An *in silico* search of the amino acid sequence of human erythrocyte catalase revealed a high relative percentage of arginine. To determine the catalase concentration in PRBC units we sampled duplicate aliquots from fresh, well-mixed PRBC bags (n=2). Aliquots were then frozen for shipment. Once at the analytical laboratory, PRBC samples were thawed at room temperature and an aliquot of around 0.5 g was hemolysed by addition of ice-cold distilled water (4.5 mL) and shaking for 3 min using a vortex mixer at room temperature. The samples were then prepared for analysis of catalase using size exclusion chromatography and inductively coupled plasma mass spectrometry (ICP-MS). After that a solution of ethanol/ chloroform (1:1), 1.5 mL per 5 mL of cell lysate, was added in order to accomplish hemoglobin precipitation. After manual shaking for 5 min, the mixture was centrifuged (12 000g, 4 °C, 1 h), and supernatant was preconcentrated using a Speed-Vac Concentrator. Following this preparation, an aliquot of 50 µl of conditioned cell lysate was injected in a size exclusion chromatography (SEC) column (Superdex^TM^ 200, 300 x 10 mm i.d, GE Healthcare Bio-Sciences Uppsala, Sweden) for separation of catalase from other iron containing molecules [[61](#_ENREF_61)] and eluted under isocratic conditions with a mobile phase of 0.05 M ammonium acetate buffer (pH 7.4) at a flow rate of 0.75 mL min^-1^. Specific atomic detection of Fe in the column effluent was performed using an ICP-MS model Thermo X series II (Thermo Scientific, Bremmen Germany) equipped with a collision cell system. H_2_ was used as collision gas (3.0 mL min^-1^) to reduce the ^40^Ar^16^O^+^ interference on ^56^Fe^+^ [[61](#_ENREF_61)]. A post-column isotope dilution analysis (IDA) of iron present in catalase was then performed using a standard solution (28 ng mL^-1^) of Fe isotopically enriched in ^57^Fe (89.768 % of ^57^Fe, 9.144 % of ^56^Fe), which was continuously pumped through a T piece situated at the end of the chromatographic column. This solution was mixed with the eluent coming from the SEC column and the mixture was introduced into the ICP-MS detector. The chromatograms obtained in the form of ^57^Fe and ^56^Fe signal intensity (counts/s) vs. time were converted, by using the online IDA calculations, into mass flow chromatograms (ng Fe vs. time) [[62](#_ENREF_62),[63](#_ENREF_63)]. From these chromatograms the amount of Fe in the chromatographic peak of catalase was obtained by integration using Origin 8. Taking into account that catalase contains four atoms of Fe per mole of protein the final concentration of catalase was calculated from the amount of Fe measured. The results obtained were corrected taking into account that 80 ± 10 % of Fe associated to catalase is recovered after the whole analytical procedure. [[61](#_ENREF_61)])
